# Anemia, iron deficiency, and blood biomarkers for Alzheimer disease: clinical interpretation and dementia risk stratification

**DOI:** 10.3389/fnut.2026.1897378

**Published:** 2026-07-16

**Authors:** Ze Li, Qiuli Ming, Linpeng Fu, Jingshuang Yang, Yilin Lv, Bingqian Chen, Xiangjun Qiu, Zhaofeng Lu

**Affiliations:** 1 The First Affiliated Hospital and College of Clinical Medicine, Henan University of Science and Technology, Luoyang, China; 2 Third Affiliated Hospital of Henan Medical University, Xinxiang, China; 3 College of Basic Medicine and Forensic Medicine, Henan University of Science and Technology, Luoyang, China

**Keywords:** Alzheimer disease, anemia, blood biomarkers, dementia risk stratification, GFAP, iron deficiency, NfL, p-tau217

## Abstract

Anemia and iron deficiency are common in older adults, yet their relevance to blood biomarkers for Alzheimer disease (AD) remains underappreciated. As these biomarkers move into clinical pathways, their interpretation depends not only on assay performance but also on comorbidity, kidney function, inflammation, and nutritional status. Against this backdrop, anemia, absolute iron deficiency, and functional iron deficiency are better viewed as distinct hematopoietic nutritional phenotypes than as background covariates, with important implications for interpreting the amyloid-β42/40 ratio, phosphorylated tau 217 (p-tau217), glial fibrillary acidic protein (GFAP), and neurofilament light chain (NfL). Current human data link anemia and iron-restricted states to higher dementia risk and to variation in plasma p-tau217, GFAP, and NfL; kidney function, body mass index (BMI), inflammation, and multimorbidity further shift biomarker distributions and cutoff interpretation. Among available analytes, p-tau217 is closest to routine clinical use within validated intended-use diagnostic pathways; however, its interpretation remains assay- and cutoff-specific and depends on pretest probability, gray-zone handling, kidney function, BMI, hemoglobin, ferritin, and transferrin saturation. Mechanistic work supports plausible links involving impaired oxygen delivery, disrupted iron–mitochondrial bioenergetics, hepcidin-mediated functional iron restriction, and glial, myelin, and blood–brain barrier vulnerability, although a complete human causal chain has not been established. Current evidence therefore favors phenotype-aware interpretation over hemoglobin-only screening or biomarker-only reading. In memory clinics and geriatric practice, complete blood count and iron studies may provide useful interpretive context in selected patients, particularly when multimorbidity, chronic kidney disease (CKD), inflammation, nutritional vulnerability, fatigue, or low or low-normal hemoglobin could affect biomarker interpretation or downstream decisions. Future studies should test whether, and in whom, correcting iron-related phenotypes changes biomarker-guided risk stratification, biomarker trajectories, or clinical decisions.

## 1 Introduction

### 1.1 Alzheimer disease blood biomarkers have changed the clinical question

Alzheimer disease (AD) blood biomarkers have shifted from a research promise to an implementation challenge, with anti-amyloid therapies and specialist pathways now requiring scalable tools to identify AD pathology in symptomatic patients before invasive or costly testing (1). Recent implementation recommendations place blood biomarkers within the broader clinical pathway, after history taking, cognitive assessment, routine bloodwork, and review of comorbidities and medications (2). The specialized-care guideline sharpens this distinction further, separating high-sensitivity triage from high-sensitivity, high-specificity confirmatory use and restricting both to patients with objective cognitive impairment who have already undergone a comprehensive memory-disorder evaluation (3).

Accordingly, the question is no longer simply whether blood biomarkers detect amyloid pathology; it is what level of performance is acceptable, who should be tested, and whether a positive result should lead to positron emission tomography (PET), cerebrospinal fluid (CSF) analysis, or treatment selection (4). Imaging and CSF biomarkers, together with cognitive assessment, counseling, and disclosure, still define the diagnostic frame, but blood tests can now reshape the sequence of specialty evaluation and eligibility assessment (5). That change has become clinically plausible with percentage-based plasma phosphorylated tau 217 (%p-tau217), whose performance in cognitively impaired cohorts is comparable with, and in some analyses exceeds, established CSF tests for detecting AD pathology (6). Once blood tests are used for triage, interpretation inevitably has to accommodate kidney function, body mass index (BMI), medication exposure, and comorbid disease; in that setting, a hematopoietic nutritional lens is more informative than a biomarker-only view (7).

### 1.2 Anemia and iron deficiency are not interchangeable, and neither should be treated as mere background variables

Anemia and iron deficiency are often compressed into the same clinical shorthand, although they refer to different biological states: anemia is defined by low hemoglobin (Hb), whereas iron deficiency reflects insufficient iron stores or bioavailable iron and may occur with or without anemia (8). Reliance on Hb alone will miss nonanemic iron deficiency, a clinically relevant state that typically requires ferritin, transferrin saturation (TSAT), or related iron studies for detection (9). In older adults and in patients with multimorbidity, ferritin must be read with particular caution, as it reflects stored iron but also tracks inflammatory activity; pairing it with TSAT yields a more interpretable picture (10). Population data underscore the point, showing that both absolute and functional iron deficiency are common in U.S. adults, including individuals without anemia, heart failure, chronic kidney disease (CKD), or pregnancy (11).

Even the label “normal” can be misleading, as many ferritin reference intervals were derived from populations or laboratory methods that may not have excluded individuals already at risk of iron deficiency (12). Chronic inflammation further complicates interpretation by increasing hepcidin signaling and restricting iron availability for erythropoiesis, thereby producing anemia of inflammation without depleted iron stores (13). For dementia risk stratification, anemia and iron deficiency are best treated as related but separable hematopoietic nutritional phenotypes, not as background covariates to be adjusted away without laboratory interpretation (14). Core definitions for anemia and iron phenotypes used throughout this Review are summarized in Table 1.

**Table 1:** Core anemia and iron phenotype definitions.

Term	Definition	Anchor	Message
Anemia	Low Hb red-cell endpoint; iron status unspecified.	Hb	Do not equate anemia with iron deficiency.
Absolute iron deficiency	Depleted iron stores, with or without anemia.	Low ferritin; often low TSAT	May precede an Hb-defined anemia threshold.
Functional iron deficiency	Insufficient usable iron despite preserved or incompletely depleted stores.	Low TSAT; ferritin normal/high with inflammation or CKD	Relevant in chronic disease, inflammation, CKD.
Ferritin	Storage iron marker.	Ferritin	High/normal values may mask iron restriction during inflammation.
TSAT	Circulating iron availability marker.	TSAT (%)	Complements ferritin for phenotype assignment.

Definitions guide phenotype assignment, not treatment thresholds. Ferritin and TSAT require inflammatory and renal context in older adults. CKD, chronic kidney disease; Hb, hemoglobin; TSAT, transferrin saturation.

### 1.3 Aim of this review: from association to a framework for stratification and interpretation

As AD is increasingly defined and staged in biological terms, a review confined to associations between anemia or iron-related markers and cognition no longer captures the clinical problem in front of us (15). In cognitively impaired patients, blood biomarkers now inform diagnosis, prognosis, and management, which means that results have to be interpreted in light of intended use and pretest probability, not as generic measures of disease burden (16). Evidence from specialized care also makes clear that performance differs substantially across blood tests, assays, and thresholds, so the statement that “blood biomarkers work” is clinically incomplete (17). The issue becomes tangible when p-tau217 cutoffs shift with kidney function, body mass index, and anemia status: the practical question is how to read an abnormal value in a specific patient (18).

Community-based evidence reaches a similar conclusion, as the predictive meaning of AD blood biomarkers changes with baseline risk, follow-up duration, and clinical setting (19). The central implementation challenge lies in embedding results within workflow, population diversity, clinician and patient education, and downstream decision making, not in assay availability alone (20). This Review moves from a catalog of associations to an interpretive model that links phenotype definition, biomarker reading, human risk evidence, and clinical translation within a single logic of dementia risk stratification (21).

### 1.4 Search strategy and selection criteria

References for this Review were identified through searches of PubMed and Web of Science for articles published from January 1, 2000, to May 1, 2026. Search terms combined Alzheimer disease and dementia (“Alzheimer disease,” “Alzheimer’s disease,” “dementia,” and “cognitive impairment”), blood biomarkers (“blood biomarker,” “plasma biomarker,” “Aβ42/40,” “p-tau217,” “p-tau181,” “p-tau231,” “GFAP,” and “NfL”), anemia and iron status (“anemia,” “anaemia,” “hemoglobin,” “haemoglobin,” “iron deficiency,” “ferritin,” “transferrin saturation,” “TSAT,” and “hepcidin”), and systemic modifiers (“kidney function,” “eGFR,” “inflammation,” “CRP,” “BMI,” and “multimorbidity”). Additional articles were identified from reference lists of relevant guidelines, consensus statements, reviews, and key original studies. Only peer-reviewed English-language publications were reviewed. Priority was given to human cohort studies, biomarker-validation and implementation studies, clinical guidelines or consensus statements, and mechanistic studies relevant to phenotype definition, biomarker interpretation, dementia risk stratification, or clinical translation. Conference-only abstracts, unpublished data, submitted manuscripts, and personal communications were excluded. The final reference list was selected on the basis of originality and relevance to the scope of this Review.

## 2 Hematopoietic nutritional phenotypes in older adults: definitions, subtypes, and interpretation

### 2.1 Clinical definitions of anemia and their limitations

Anemia is still defined by Hb in most clinical and public health settings, with thresholds interpreted according to age, sex, pregnancy status, and measurement context (22). That simplicity has clear value for surveillance and severity grading, as illustrated by burden estimates tracking anemia prevalence, years lived with disability, and cause-specific patterns across populations (23). At the same time, recent pooled reference analyses challenge the assumption that inherited cutoffs are immutable, reporting 5th percentile Hb thresholds in healthy populations that do not fully align with traditional World Health Organization (WHO) values (24). In older adults, Hb remains necessary for case definition, but it is only the entry point: anemia at this age may reflect nutritional deficiency, chronic inflammation, kidney disease, occult blood loss, clonal hematopoiesis, or hematologic malignancy (25).

A further limitation is that a substantial share of late-life anemia remains unexplained after routine evaluation, underscoring how little the label “anemia” reveals about underlying biology (26). Gerontology literature accordingly continues to treat unexplained anemia of aging as a diagnosis of exclusion, with mechanisms, trajectory, and clinical consequences still only partly resolved (27). Hb should mark the boundary of anemia, but interpretation has to extend beyond that boundary to morphology, reticulocyte response, kidney function, inflammation, and iron-related testing before the result enters downstream modeling or clinical inference (28).

### 2.2 Absolute versus functional iron deficiency

Absolute iron deficiency refers to depleted body iron stores, whereas functional iron deficiency describes preserved or incompletely depleted stores that cannot be mobilized fast enough to sustain erythropoiesis; the distinction concerns iron availability, not merely severity along a single continuum (29). The laboratory logic follows the same division: ferritin primarily reflects storage, whereas TSAT reflects circulating availability, and ferritin alone becomes difficult to interpret when inflammation, multimorbidity, or acute-phase responses elevate it independently of usable iron (10). Mechanistically, functional iron deficiency is rooted in the hepcidin–ferroportin axis, where increased hepcidin suppresses intestinal absorption and macrophage iron release, producing iron-restricted erythropoiesis despite retained storage iron (30). Clinical algorithms for chronic disease often define iron deficiency by TSAT <20% alongside disease-specific ferritin ranges, acknowledging that heart failure, CKD, inflammatory bowel disease, and cancer can raise ferritin while still limiting iron delivery to marrow (31).

These phenotypes are not rare edge cases. National Health and Nutrition Examination Survey (NHANES) 2017–2020 data estimated absolute iron deficiency in 14% and functional iron deficiency in 15% of U.S. adults, with both states present even among individuals without anemia, heart failure, CKD, or pregnancy (11). Older adults are especially susceptible to mixed patterns, as occult gastrointestinal blood loss, poor intake, impaired absorption, CKD, obesity, frailty, and chronic low-grade inflammation can move the same patient between depleted stores and restricted availability over time (32). Clinical descriptions of iron deficiency have accordingly shifted toward a spectrum model, in which iron depletion, impaired erythropoiesis, anemia, and nonanemic symptoms do not necessarily unfold in a single linear sequence from low ferritin to anemia (33). In this Review, absolute and functional iron deficiency are handled as explicitly defined hematopoietic nutritional phenotypes, not as Hb surrogates or reassuring “normal ferritin” labels that bypass the biology of iron availability (34).

### 2.3 Ferritin, TSAT, CRP, hepcidin, and eGFR: how to interpret them in older adults

In older adults, iron studies are best interpreted from clinical context outward, since inflammation and chronic disease can mask iron deficiency when conventional low-ferritin rules are applied; in a multicenter geriatric-unit study, ferritin <100 μg/L and/or TSAT <20% identified iron deficiency as common even in patients without anemia (35). Ferritin remains informative as a storage marker, but any conclusion drawn from it is highly cutoff-dependent: in Swiss primary care, thresholds of 15, 30, and 45 ng/mL yielded markedly different diagnostic rates for iron deficiency (36). The same instability appears at population level, where the Hemochromatosis and Iron Overload Screening Study, WHO, and iron-deficient erythropoiesis definitions produced substantially different prevalence estimates among women in the United States and Canada (37).

TSAT captures circulating iron availability and becomes especially useful when CKD, inflammation, or other chronic disease states elevate ferritin (38). C-reactive protein (CRP) does not define iron deficiency, but it helps determine whether ferritin still behaves as a storage proxy or whether a low-TSAT/high-ferritin pattern is more consistent with iron-restricted erythropoiesis (39). Hepcidin sits closer to mechanism than to routine screening; it may clarify mixed iron deficiency anemia, anemia of chronic disease, or overlapping states, but current evidence supports selective use instead of broad first-line testing (40).

Renal function belongs in the same interpretive frame, as reduced estimated glomerular filtration rate (eGFR) can alter hepcidin handling and make the ferritin–hepcidin relationship less stable in the setting of inflammation or kidney dysfunction (41). In older adults, ferritin, TSAT, CRP, and eGFR are most informative when read as a contextual panel; the Kidney Disease: Improving Global Outcomes guideline formalizes this approach in CKD by recommending complete blood count (CBC), reticulocytes, ferritin, and TSAT as core components of anemia and iron evaluation (42).

### 2.4 Why hemoglobin alone is insufficient

Hb remains indispensable for defining anemia, but it is too distal a red-cell endpoint to stand in for iron stores or iron availability; normal Hb can coexist with nonanemic iron deficiency when symptoms, risk factors, or laboratory context point to depleted or restricted iron biology (43). Consensus recommendations make this boundary explicit, advising against the use of Hb or hematocrit alone to identify iron deficiency and emphasizing that ferritin-based assessment still requires clinical context and, when necessary, additional biomarkers (44). The consequence is not trivial: among nonpregnant U.S. adults, broadening ferritin criteria from ≤15 to ≤30 or ≤45 ng/mL while keeping WHO anemia thresholds unchanged substantially increased the estimated prevalence of iron deficiency anemia (45).

Normal Hb is equally poor as a proxy for iron repletion, since ferritin distributions among working-age adults without anemia vary by sex and age, separating the iron-store boundary from the anemia boundary in biological terms (46). Interpretation is further complicated by inconsistency in ferritin reference intervals across laboratories, so a value reported as “normal” may reflect local conventions more than a stable decision limit (47). Reticulocyte-derived parameters can detect iron-restricted erythropoiesis earlier than mature erythrocyte indices, whereas Hb often falls only after marrow iron delivery has already been compromised (48).

Clinical relevance also extends beyond the anemia label, with recent evidence syntheses linking nonanemic iron deficiency to fatigue, reduced quality of life, and cognitive or psychiatric symptom domains, albeit in literature broader than geriatric cognition alone (49). Hb should mark the red-cell endpoint; iron phenotype assignment should rest on ferritin, TSAT, and context-specific decision limits before results are taken forward into downstream interpretation (50). Key laboratory definitions are summarized in Table 1, and phenotype-first interpretive points are summarized in Table 2.

**Table 2:** Phenotype-first laboratory interpretation.

Source	Setting	Phenotype	Rule	Message	Caveat	Refs.
Guideline consensus	Broad ID/IDA contexts	Anemia; absolute ID; functional ID; ferritin; TSAT	Hb for anemia; ferritin <30 μg/L without inflammation; TSAT in inflammatory states	Assign phenotype; avoid Hb-only reasoning	Not dementia-specific	(10)
Guideline	CKD, dialysis, kidney transplant	CKD anemia; systemic ID; iron-restricted erythropoiesis	CBC, reticulocytes, ferritin, TSAT	Renal context shapes iron interpretation	CKD terminology	(42)
Expert recommendations	Adults, pregnancy, children	ID with or without anemia; ferritin-based assessment	Avoid Hb/hematocrit-only diagnosis; interpret ferritin in context	Context defines iron status	No geriatric cutoff	(44)
Cross-sectional study	U. S. adults, NHANES	Absolute ID; functional ID; ferritin; TSAT	Absolute ID: ferritin <30 ng/mL; functional ID: ferritin ≥30 ng/mL plus TSAT <20%	Functional ID can be Hb-normal	Cross-sectional	(11)
Retrospective cohort	Swiss primary care adults	Ferritin-cutoff ID; ferritin; Hb; CRP	Ferritin 15, 30, or 45 ng/mL	Cutoff choice changes classification	No TSAT/hepcidin validation	(36)
Cross-sectional study	U. S. nonpregnant adults	IDA by ferritin and Hb	Ferritin ≤15, ≤30, or ≤45 ng/mL plus WHO Hb thresholds	IDA prevalence depends on rules	IDA only	(45)
Pooled reference analysis	Healthy adults 18–65 y	Hb thresholding	5th percentile Hb: men 134.9 g/L; women 119.7 g/L	Hb defines anemia, not iron phenotype	No adults >65 y	(24)
Cross-sectional study	U. S./Canada women	Ferritin- and TSAT-defined ID	TSAT <10% plus ferritin <15 ng/mL versus ferritin-only thresholds	Definition changes ID prevalence	Women only	(37)

Laboratory rules support phenotype assignment in clinical context, not treatment recommendations. CBC, complete blood count; CKD, chronic kidney disease; CRP, C-reactive protein; Hb, hemoglobin; ID, iron deficiency; IDA, iron deficiency anemia; NHANES, National Health and Nutrition Examination Survey; TSAT, transferrin saturation; WHO, World Health Organization.

## 3 The current landscape of Alzheimer disease blood biomarkers and why hematopoietic status matters for interpretation

### 3.1 What Aβ42/40, p-tau181/217/231, GFAP, and NfL actually capture

Plasma biomarkers relevant to AD are not interchangeable signals. The amyloid-β42/40 ratio (Aβ42/40), phosphorylated tau species, glial fibrillary acidic protein (GFAP), and neurofilament light chain (NfL) map onto partially overlapping but biologically distinct dimensions of amyloid accumulation, AD-related tau response, astroglial activation, and neuroaxonal injury (51). Aβ42/40 is the plasma measure most closely aligned with cerebral amyloid state, shifting early, remaining relatively low once abnormal, and helping identify risk of cognitive decline across clinically unimpaired and cognitively impaired stages, even if its concentration change is smaller than that of many downstream markers (52). Plasma phosphorylated tau 181 (p-tau181) captures an AD-related tau-phosphorylation response that becomes increasingly informative along the clinical continuum and can assist in distinguishing AD from non-AD dementias, but it is best regarded as an analyte-specific tau signal, not a direct measure of total tau (t-tau) burden (53).

Within the plasma tau analytes, phosphorylated tau 217 (p-tau217) currently offers the readout most closely aligned with biological AD, showing high diagnostic accuracy for abnormal amyloid and tau pathology across independent cohorts and wider separation between AD and non-AD groups than many earlier plasma tau measures (54). Phosphorylated tau 231 (p-tau231) appears to mark an earlier, amyloid-responsive phase of tau biology, with sensitivity to the first cerebral amyloid-*β* (Aβ) changes before overt plaque positivity becomes the dominant reference point (55). GFAP, by contrast, primarily reflects astrocytic reactivity; in cognitively unimpaired older adults, higher plasma levels have been linked to amyloid-related risk, cognitive decline, cortical atrophy, and later progression, but the marker is better read as a glial-response signal than as a direct proxy for tau pathology (56).

NfL adds a different dimension, reflecting neuroaxonal injury and broader neurodegeneration severity, which gives it prognostic value but limits its specificity for AD pathology (57). The p-tau family is also internally heterogeneous: p-tau181, p-tau217, and p-tau231 all carry AD-related information, yet they differ in timing, dynamic range, and diagnostic discrimination, so one result should not be substituted mechanically for another (58). Early-detection cohort data support a panel-based view in which plasma biomarkers provide partially overlapping, not fully redundant, information; the practical issue is marker readiness, not simple interchangeability (59).

### 3.2 Which biomarkers are closest to clinical use

At present, p-tau217-based testing is closest to routine use within validated intended-use pathways, whereas Aβ42/40, GFAP, and NfL more often serve as complementary markers for staging, prognosis, or context-sensitive interpretation (60). Throughout this Review, p-tau217 refers to the analyte concentration, whereas %p-tau217 refers to a percentage-based assay format reported in specific studies and should not be used interchangeably with raw p-tau217 values. In symptomatic patients from Swedish primary and secondary care, %p-tau217 and amyloid probability score 2 (APS2) achieved high diagnostic accuracy with predefined cutoffs, positioning p-tau217-centered testing as one of the most mature options for real-world triage (61). A fully automated plasma p-tau217 assay has shown similarly strong performance across European secondary-care cohorts and Swedish primary care, bringing the marker closer to routine laboratory workflows (62).

Ratio strategies may reduce uncertainty further. In Chinese clinical and community cohorts, plasma p-tau217/Aβ42 achieved high PET-based accuracy and yielded a smaller intermediate zone than p-tau217 alone (63). The status of p-tau217 is reinforced by data showing diagnostic performance broadly comparable with CSF p-tau217 in biomarker-defined AD (64). High-throughput immunoassay studies extend this evidence beyond binary detection to amyloid selection and tau-stage estimation, strengthening the case for calibrated p-tau217 implementation in defined clinical pathways (65).

Field testing in memory clinics supports the same hierarchy, while also underscoring its limits: plasma biomarker pathways can improve diagnostic efficiency, but not every analyte in a panel is ready for stand-alone clinical decisions (66). For the moment, within validated intended-use pathways for symptomatic or objectively cognitively impaired patients, p-tau217-centered assays are best viewed as the most clinically mature blood-biomarker option; p-tau217/Aβ42 or related ratios may help reduce uncertainty, whereas Aβ42/40, GFAP, and NfL remain supportive analytes whose value depends on setting, comorbidity, assay platform, and downstream confirmation (67). This hierarchy supports calibrated use in defined pathways, not population screening or assay-interchangeable thresholds.

### 3.3 How kidney function, BMI, inflammation, and anemia modify biomarker readings

Clinical readiness does not remove context dependence. Plasma biomarkers relevant to AD reflect more than AD pathology, and the same concentration can mean different things when age, sex, BMI, kidney disease, vascular disease, and other chronic conditions alter the background against which it is interpreted (68). Renal function provides the clearest example: CKD stage 3b or worse has been associated with higher plasma p-tau217 and with movement of some amyloid-negative individuals toward false-positive classification, whereas %p-tau217 appears less sensitive to this effect (69). Even this renal influence is not uniform across cohorts, as eGFR shifted several biomarker concentrations in Translational Biomarkers in Aging and Dementia (TRIAD) but contributed little to p-tau-based prediction of amyloid positivity in a cohort with predominantly normal-to-mild kidney impairment (70).

BMI introduces a different interpretive problem. Higher BMI and larger blood volume have been associated with lower plasma p-tau217, GFAP, and NfL, and this dilution effect can alter threshold-based amyloid PET classification even when brain amyloid burden is unchanged (71). Community-based data add further complexity: multimorbidity, anemia, kidney disease, heart disease, cerebrovascular disease, diabetes, BMI, and interleukin-6 (IL-6) were all associated with variation in serum Aβ42/40, p-tau181, NfL, and GFAP among older adults, placing hematologic and inflammatory status squarely inside the interpretive field (72). Similar renal associations were observed in a Hispanic/Latino community sample, where CKD status, eGFR, and albuminuria remained linked to plasma amyloid/tau/neurodegeneration (ATN) markers after adjustment for cardiometabolic factors (73).

These effects are not merely statistical nuisances: nutritional deficiency, systemic inflammation, and metabolic dysregulation may shift blood biomarker levels through pathways outside the canonical amyloid–tau sequence (74). Clinically usable interpretation therefore requires kidney function, BMI, inflammatory burden, anemia, and local population context to be considered before fixed cutoffs are translated into dementia risk stratification (75).

### 3.4 From marker performance to context-aware interpretation

Average areas under the curve, sensitivity, and specificity are essential for judging AD blood biomarkers, but they do not determine where cutoffs should sit, how gray-zone values should be handled, which patients should be tested, or how results should be integrated into workflow (76). The same p-tau217 value can yield very different positive or negative predictive value depending on age, apolipoprotein E (APOE) ε4 status, clinical syndrome, and pretest probability, so interpretation has to be anchored to the patient’s prior likelihood of amyloid pathology (77). Cutoff studies make this practical: a single p-tau217 threshold can project more certainty than the data support, whereas two-cutoff or three-zone strategies better separate rule-out, intermediate, and rule-in ranges (78).

Bayesian implementation brings the same logic into real-world memory clinics by combining clinician-estimated pretest probability with the likelihood ratio generated by plasma p-tau217, thereby converting a laboratory value into an individualized posttest probability (79). Even a blood test with strong clinical validity against amyloid PET or CSF remains useful only within the right intended population, reference standard, assay calibration, and strategy for handling intermediate results (80). Clinical utility sets the bar higher still: the test has to change diagnostic confidence, referral patterns, confirmatory PET or CSF use, disclosure, or management, not merely reproduce a reference-standard classification (81). Accordingly, intermediate-zone p-tau217 results should be treated as unresolved evidence requiring clinical integration, repeat testing, longitudinal follow-up, or confirmatory PET/CSF rather than as stand-alone positive or negative diagnoses.

**Figure 1:**
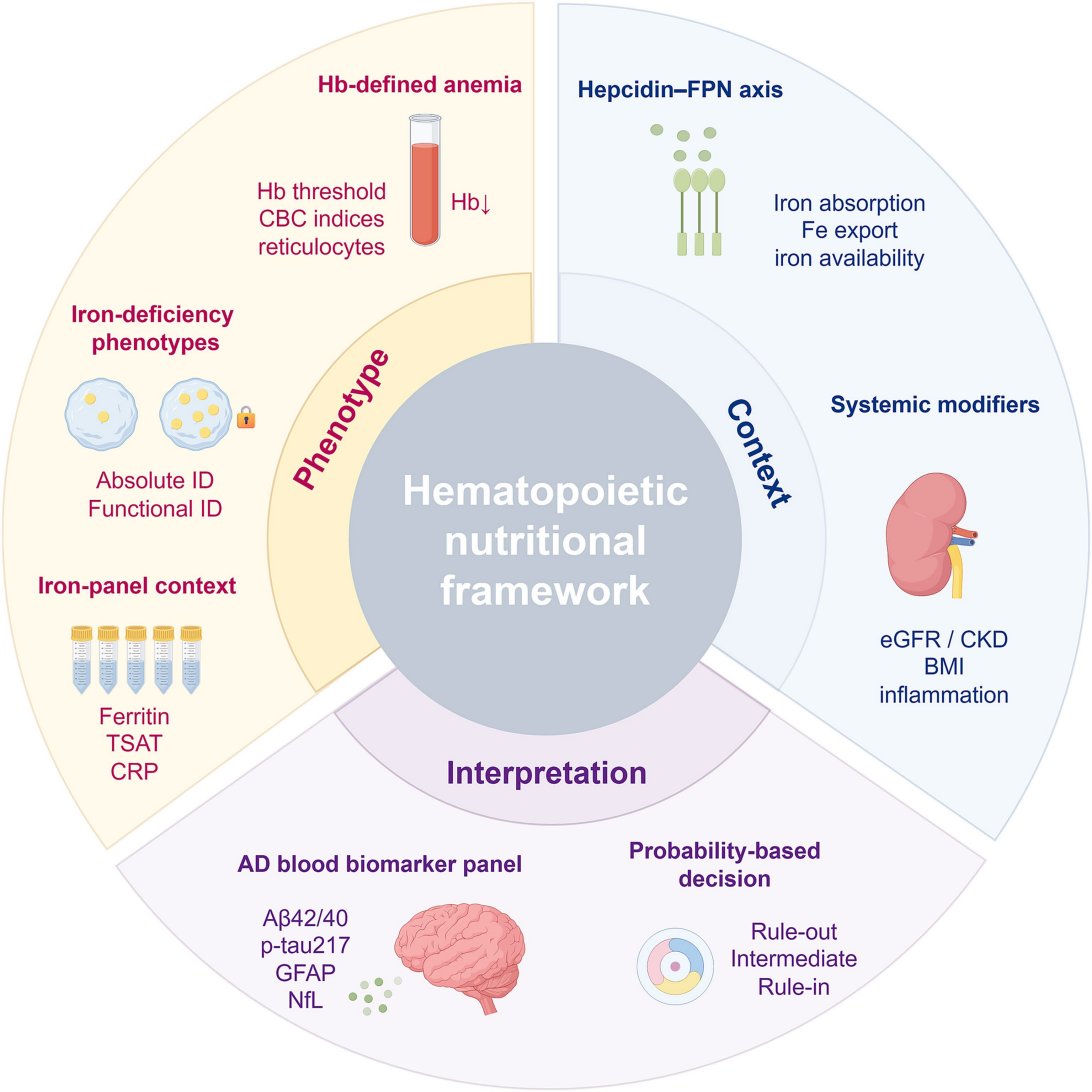
Hematopoietic nutritional framework for context-aware interpretation of Alzheimer disease blood biomarkers. Created by Figdraw.com.

Generalizability is part of the same problem, as community-based and diverse biomarker datasets are needed to determine whether performance and thresholds travel across recruitment settings, racial and ethnic groups, comorbidity burden, and health care access (82). AD blood biomarkers are better treated as conditional evidence whose meaning depends on analyte biology, assay platform, cutoff strategy, clinical probability, non-AD modifiers, and the care pathway in which the result is being used (83) (Figure 1). Major AD blood biomarkers and their principal non-AD modifiers are summarized in Table 3.

**Table 3:** AD blood biomarkers and non-AD modifiers.

Setting	Biomarkers	Context	Modifiers	Message	Caveat	Ref.
Swedish primary and secondary care	%p-tau217; APS2	AD triage	Care level	High accuracy in symptomatic care	Non-AD modifiers not tested	(61)
European secondary care; Swedish primary care	Automated p-tau217; p-tau217/Aβ42; %p-tau217	One- and two-cutoff diagnosis	Age; CKD; diabetes; sex; APOE	Two cutoffs and ratios reduce uncertainty	No direct BMI/anemia modeling	(62)
TRIAD, WRAP, SPIN	Plasma p-tau217	Amyloid/tau detection	None modeled	Strong pathology anchor	No modifier analysis	(54)
11 cognitively impaired cohorts	p-tau217; p-tau181; p-tau231; GFAP; NfL	Amyloid probability modeling	Age; APOE ε4; syndrome; pretest probability	Pretest probability changes posttest meaning	Model-based	(77)
ADNI	Aβ42/40; p-tau181; p-tau217; GFAP; NfL	Assay comparison	Age; sex; APOE	p-tau217 strongest across AD outcomes	Research cohort	(51)
Mayo MCI or mild dementia cohorts	ALZpath and Lumipulse p-tau217 assays	Amyloid PET cutoffs	Assay-specific cutoffs	Two-cutoff workflows reflect uncertainty	Mild symptomatic only	(78)
Mayo CKD-enriched cohorts	p-tau217; %p-tau217	Renal confounding	eGFR; CKD stage; amyloid status	CKD affects raw p-tau217; %p-tau217 less affected	Limited severe CKD	(69)
TRIAD	Aβ42/40; p-tau species; GFAP; NfL	Kidney effects	eGFR; CKD	eGFR shifts levels more than prediction	Mostly mild kidney impairment	(70)
Community older adults	Aβ42/40; p-tau181; t-tau; NfL; GFAP; IL-6	Community variation	Multimorbidity; anemia; CKD; CVD; diabetes; BMI; IL-6	Systemic burden shifts background levels	No PET/CSF; cross-sectional	(72)
ADNI cognitively unimpaired	Aβ42/40; p-tau181; p-tau217; GFAP; NfL	BMI and blood-volume effects	BMI; blood volume	Higher BMI/blood volume lowers concentrations	Preclinical cohort	(71)
IGNITE cognitively unimpaired older adults	Aβ42/40; p-tau181; p-tau217; p-tau217/Aβ42; GFAP; NfL	Early Aβ detection	Diabetes; CVD; BMI; comorbidity	Cardiometabolic burden weakens early Aβ detection	Small disease-positive subgroups	(111)
Chinese clinic and community cohorts	Lumipulse p-tau217; p-tau217/Aβ42; p-tau181; p-tau181/Aβ42; Aβ42/40	PET-based detection	Setting	p-tau217/Aβ42 reduces intermediate zone	Chinese-only cohorts	(63)

Interpretation requires assay-specific calibration and intended-use context. AD, Alzheimer disease; ADNI, Alzheimer’s Disease Neuroimaging Initiative; APOE, apolipoprotein E; APS2, amyloid probability score 2; Aβ, amyloid-β; Aβ42/40, amyloid-β42/40 ratio; BMI, body mass index; CKD, chronic kidney disease; CSF, cerebrospinal fluid; CVD, cardiovascular disease; eGFR, estimated glomerular filtration rate; GFAP, glial fibrillary acidic protein; IGNITE, Investigating Gains in Neurocognition in an Intervention Trial of Exercise; IL-6, interleukin-6; MCI, mild cognitive impairment; NfL, neurofilament light chain; PET, positron emission tomography; p-tau, phosphorylated tau; %p-tau217, percentage-based plasma phosphorylated tau 217; SPIN, Sant Pau Initiative on Neurodegeneration; TRIAD, Translational Biomarkers in Aging and Dementia; t-tau, total tau; WRAP, Wisconsin Registry for Alzheimer’s Prevention.

Anemia, absolute iron deficiency, and functional iron deficiency are proposed as contextual phenotypes to be considered alongside ferritin, TSAT, CRP, hepcidin–FPN signaling, renal function, BMI, and inflammatory burden when AD blood biomarker results are translated into rule-out, intermediate, or rule-in decisions. The figure was created using Figdraw. AD, Alzheimer disease; A*β*, amyloid-β; Aβ42/40, amyloid-β42/40 ratio; BMI, body mass index; CBC, complete blood count; CKD, chronic kidney disease; CRP, C-reactive protein; eGFR, estimated glomerular filtration rate; FPN, ferroportin; GFAP, glial fibrillary acidic protein; Hb, hemoglobin; ID, iron deficiency; NfL, neurofilament light chain; p-tau217, phosphorylated tau 217; TSAT, transferrin saturation.

## 4 Human evidence linking anemia, iron deficiency, blood biomarkers, and dementia risk

### 4.1 Anemia and incident dementia: from association to joint-risk states

Late-life anemia is best regarded as a heterogeneous exposure, not a single biological entity, since older adults within the same population may have nutritional anemia, anemia of chronic disease, kidney-related anemia, inflammatory anemia, or anemia of uncertain origin (84). Large prospective data support an overall association: in 313,448 UK Biobank participants, anemia and several blood-cell indices were associated with higher incident dementia risk over long-term follow-up (85). Population-based aging cohorts point in the same direction while also showing why a single anemia label is too coarse, given the contributions of hypoproliferation, chronic disease, renal dysfunction, and other geriatric factors to both etiology and outcome (86).

The signal becomes more informative when inflammatory context is retained in the analysis, with UK Biobank data showing stronger associations of anemia with cognitive decline and dementia risk among participants with higher CRP (87). Longitudinal evidence from middle-aged and older Chinese adults adds an important caution: anemia was associated with later cognitive performance, but lower baseline cognition also predicted subsequent anemia, arguing against a simple one-way causal interpretation (88).

The clearest evidence for a joint-risk state comes from older adults in whom both hematologic measures and AD biomarkers were available: anemia was associated with higher baseline p-tau217, NfL, and GFAP, and dementia hazards were greatest when anemia coexisted with elevated blood biomarkers (89). Clinical dementia data beyond Alzheimer-specific cohorts similarly situate anemia within broader geriatric vulnerability, supporting a move away from treating it as a background covariate and toward reading it as part of a combined risk profile (90).

### 4.2 Anemia and p-tau217, GFAP, and NfL: why combined interpretation matters

Datasets that included Aβ42/40, p-tau217, GFAP, and NfL show that Hb and eGFR are linked to variability in p-tau217, GFAP, and NfL through largely Aβ-independent pathways, whereas APOE and BMI exert more Aβ-dependent effects; biomarker elevations in an anemic or renally vulnerable patient should not be read as direct surrogates of AD pathology alone (91). The strongest population-level evidence comes from dementia-free older adults in the Swedish National Study on Aging and Care in Kungsholmen, where anemia was associated with higher baseline p-tau217, NfL, and GFAP as well as higher incident dementia risk, and where the highest risk was seen when anemia coincided with elevated biomarkers (89). Interpreting such results also requires analyte-specific weighting: plasma p-tau217 yielded the clearest disease-stratification signal, GFAP tracked more closely with tau progression and astroglial response, and NfL showed weaker linkage to typical AD pathology and cognition, so anemia-associated elevations in these markers should not be assigned the same pathological meaning (92).

Longitudinal panel data sharpen this distinction. In cognitively unimpaired and mild cognitive impairment (MCI) populations, the combination of p-tau217, GFAP, and NfL predicted domain-specific cognitive decline better than isolated markers, with elevated NfL seeming to amplify the decline already flagged by p-tau217 and GFAP while offering limited AD specificity on its own (93). Findings from an unselected neuropsychiatry memory clinic point in the same direction: p-tau217 separated AD from behavioral variant frontotemporal dementia and primary psychiatric disorders with strong accuracy, whereas NfL was more helpful for distinguishing neurodegenerative from psychiatric presentations and GFAP added limited diagnostic value (94). Imaging-linked data reinforce the same message, showing that p-tau217 and GFAP track different segments of the ATN cascade across Centiloid scales; viewed in that light, anemia is best handled as a modifier of combined biomarker interpretation, not as grounds either to dismiss abnormal results or to accept them uncritically (95).

### 4.3 Absolute and functional iron deficiency: moving beyond hemoglobin

Human evidence moves beyond Hb once absolute and functional iron deficiency are examined directly. In the Apolipoprotein-related Mortality Risk Study, the separation of depleted stores from restricted iron availability showed that both phenotypes were associated with incident dementia, including analyses not contingent on overt anemia (96). The exposure itself is broader than laboratory phenotypes: among older UK Biobank participants, patterns of iron intake were linked to later dementia risk, suggesting that iron-related risk cannot be inferred solely from whether Hb crosses an anemia threshold or from a single laboratory value (97). Similar signals appear in older community-dwelling adults at high cardiovascular risk, where dietary iron and anemia-related markers were associated with cognition and quality of life, placing iron status within a broader functional phenotype (98). Ferritin complicates this picture, as NHANES data suggested an approximate U-shaped relation between body iron status and Digit Symbol Substitution Test (DSST) performance, with higher ferritin linked to poorer task performance after adjustment for demographic, inflammatory, and vascular factors (99). Lower serum ferritin has likewise been associated with worse cognitive performance in aging cohorts, supporting the relevance of depleted iron stores while underscoring that ferritin alone does not define the phenotype without TSAT, inflammation, and clinical context (100).

Serum iron carries a different signal from ferritin: in a rural northern China cohort, moderate or higher serum iron was associated with lower odds of cognitive impairment than low iron status among community-dwelling older adults (101). Iron-related associations also extend into vascular vulnerability, with serum multi-trace-element profiles that included iron-related measures linked to post-stroke cognitive impairment in a prospective observational cohort (102). Clinical prediction models for vascular cognitive impairment have incorporated serum iron-metabolism indicators together with cerebral microbleeds, but these models are more appropriately regarded as risk tools than as validated definitions of absolute or functional iron deficiency (103). Evidence from nonanemic iron deficiency, although not specific to aging, further weakens Hb-only reasoning: youths without anemia showed reduced basal ganglia iron content and worse neuropsychological or psychiatric measures despite the absence of a low-Hb phenotype (104).

The opposite end of iron biology also deserves attention. Hemochromatosis genotypes were associated with incident dementia in older adults, arguing against any simple “less iron, more risk” model or a monotonic reading of iron biology across the life course (105). Intervention data add a second caution: in biomarker-defined early AD, deferiprone altered hippocampal iron measured by quantitative susceptibility mapping but did not establish cognitive benefit, making iron lowering an inadequate surrogate for dementia prevention on its own (106). Nor is central iron deposition interchangeable with peripheral iron deficiency, as cerebral small vessel disease data linked brain iron deposition, plasma neurodegenerative proteins, and cognition without making ferritin, serum iron, or TSAT equivalent to brain iron (107). Taken together, current human evidence supports moving beyond Hb to explicit iron phenotyping, but it still does not justify a single dementia-risk threshold that can unify dietary iron, ferritin, serum iron, TSAT, functional deficiency, and brain iron into one clinically interpretable exposure for stratification, biomarker reading, monitoring, or intervention selection (108).

### 4.4 Stratifiers and modifiers: sex, multimorbidity, inflammation, and kidney disease

These associations are not uniform across populations, and several stratifiers alter both their strength and their meaning. Inflammation is one of the clearest: UK Biobank data showed stronger associations of anemia with poorer cognitive function and dementia among older adults with higher CRP, making inflammatory status part of the exposure context and not merely an adjustment variable (87). Sex and life stage add another layer, as systemic iron status was associated with cognitive performance in perimenopausal women even in the absence of overt anemia or clinically defined iron deficiency, suggesting that subanemic iron variation may carry different implications across sex- and life-stage-specific contexts (109). Multimorbidity also reshapes the background against which biomarkers are interpreted, with distinct multimorbidity patterns in cognitively unimpaired community-dwelling older adults associating with AD blood biomarkers more strongly than disease count alone (110).

Medical conditions may alter test performance as well as baseline risk. In Investigating Gains in Neurocognition in an Intervention Trial of Exercise, diabetes, cardiovascular conditions, BMI, and comorbidity burden weakened the diagnostic accuracy of plasma p-tau217 and p-tau217/Aβ42 for early Aβ detection (111). Kidney function warrants similar attention, as eGFR and CKD shifted several plasma biomarkers relevant to AD, although the incremental value of renal measures for predicting amyloid positivity differed across cohorts (70). Community data extend this caution by linking kidney function to circulating dementia-related biomarkers without establishing a one-to-one translation into incident dementia risk, supporting the view that kidney disease can act simultaneously as a clinical comorbidity and as a modifier of readout interpretation (112).

The same principle extends to multimorbidity more broadly. Large-scale proteomic analyses show that multimorbidity carries both shared and disease-specific blood biomarker signatures, including inflammatory and metabolic patterns that can reshape the systemic context in which dementia-related assays are interpreted (113). Current evidence thus favors stratified interpretation of hematopoietic phenotypes and blood biomarkers, but heterogeneous definitions, cohorts, and platforms still preclude fixed subgroup-specific rules; the next step is less about adding yet another association and more about defining the limits of what current human data can support (114).

### 4.5 What current human data can and cannot tell us

Current human evidence supports graded associations and phenotype-aware interpretation, but it does not yet justify treating anemia or iron-related phenotypes as established targets for dementia prevention. In the Vitamin D3–Omega-3–Home Exercise–Healthy Ageing and Longevity Trial, higher Hb quintiles were associated with lower 3-year odds of MCI, whereas the binary anemia variable was less informative in a generally healthy cohort with low anemia prevalence (115). Cross-national findings from the Longitudinal Aging Study in India–Diagnostic Assessment of Dementia and the Health and Retirement Study extend the signal beyond Europe, showing that lower Hb and anemia were associated with poorer cognitive performance among older adults in India and the United States (116). Genetic triangulation adds caution, not closure: UK Biobank Mendelian randomization linked higher TSAT to non-Alzheimer and vascular dementia (VaD) risk, which does not support a one-directional iron-deficiency-to-AD pathway (117). Even within Mendelian randomization, causal claims remain limited by instrument validity, bias assessment, and replication, so genetic evidence can refine the debate but cannot settle it (118).

Prevention implications remain plausible but unsettled. Commentary on anemia and dementia, the 2024 Lancet Commission, and umbrella-review evidence all underscore that prevention targets require convergent observational, mechanistic, and intervention evidence, not association alone (119–121). At present, human data are strongest for risk stratification, phenotype-aware interpretation, and hypothesis generation; intervention claims require repeated measurements, mechanistic biomarkers, and trial endpoints before hematopoietic nutritional correction can be linked to dementia prevention or biomarker-trajectory change (122). Representative human studies linking anemia, iron deficiency, blood biomarkers, and dementia risk are summarized in Table 4.

**Table 4:** Human evidence linking hematopoietic phenotypes and dementia risk.

Population	Exposure	Outcome	Signal	Modifiers	Caveat	Refs.
SNAC-K dementia-free older adults	Hb; anemia; p-tau217; NfL; GFAP	Incident dementia; baseline biomarkers	Anemia linked to higher biomarkers and dementia risk	Sex; APOE ε4; baseline MCI	Mostly normocytic anemia	(89)
AMORIS adults aged ≥50 years	Absolute ID; functional ID	Incident dementia	Both ID phenotypes linked to higher dementia risk	Sex; age; CVD; CCI; anemia status	Register-based diagnosis	(96)
Korean clinic cohort across cognitive stages	Hb; eGFR; BMI; APOE; CKD; Aβ42/40; p-tau217; GFAP; NfL	Biomarker variability; Aβ PET positivity	Hb/eGFR shifted p-tau217, GFAP, and NfL independent of Aβ	Aβ-independent and Aβ-dependent pathways	No incident dementia outcome	(91)
UK Biobank	Anemia; Hb; blood-cell indices	Incident dementia; AD; VaD	Anemia/lower Hb linked to higher dementia risk	Age; sex; APOE ε4; subtype	Not an iron-phenotype study	(85)
DO-HEALTH healthy older adults	Hb quintiles; anemia	3-year MCI	Higher Hb quintiles linked to lower MCI odds	Sex; age; baseline iron status	Healthy cohort; low anemia prevalence	(115)
LASI-DAD and HRS	Anemia; Hb; blood indices	Cross-sectional cognition	Lower Hb/anemia linked to poorer scores	Cross-national replication; sex	Cross-sectional	(116)
NHANES older adults	Serum ferritin	Cross-sectional DSST	Approximate U-shaped cognitive association	Age; CRP; renal/stroke sensitivity analyses	Ferritin alone insufficient	(99)
Rural northern China older adults	Serum iron	Cross-sectional cognitive impairment	Moderate/high iron linked to lower impairment odds than low iron	Age; education; BMI; lifestyle; diabetes; dyslipidemia	No ferritin/TSAT/Hb panel	(101)

Studies are observational and should not be read as causal evidence. AD, Alzheimer disease; AMORIS, Apolipoprotein-related Mortality Risk Study; APOE, apolipoprotein E; Aβ, amyloid-β; Aβ42/40, amyloid-β42/40 ratio; BMI, body mass index; CCI, Charlson Comorbidity Index; CKD, chronic kidney disease; CRP, C-reactive protein; CVD, cardiovascular disease; DO-HEALTH, Vitamin D3–Omega-3–Home Exercise–Healthy Ageing and Longevity Trial; DSST, Digit Symbol Substitution Test; eGFR, estimated glomerular filtration rate; GFAP, glial fibrillary acidic protein; Hb, hemoglobin; HRS, Health and Retirement Study; ID, iron deficiency; LASI-DAD, Longitudinal Aging Study in India–Diagnostic Assessment of Dementia; MCI, mild cognitive impairment; NfL, neurofilament light chain; NHANES, National Health and Nutrition Examination Survey; PET, positron emission tomography; p-tau217, phosphorylated tau 217; SNAC-K, Swedish National Study on Aging and Care in Kungsholmen; TSAT, transferrin saturation; VaD, vascular dementia.

To make explicit how different types of evidence are used in this Review, Box 1 separates human association evidence, biomarker-modifier evidence, mechanistic plausibility, and practice implications before moving to the mechanistic literature.

Box 1 **Evidence hierarchy used in this review.**

**Human association evidence.** Cohort and population studies link anemia, lower Hb, and iron-deficient or iron-restricted phenotypes with cognitive outcomes or incident dementia. These data support risk stratification and hypothesis generation rather than causal inference about AD.

**Biomarker-modifier evidence.** Hb, anemia status, kidney function, BMI, inflammation, and multimorbidity can shift plasma p-tau217, GFAP, NfL, Aβ42/40, or related biomarker distributions. These data support context-aware interpretation, gray-zone handling, and decisions about confirmatory PET or CSF, without automatically reclassifying abnormal AD biomarkers as non-AD signals.

**Mechanistic plausibility.** Hypoxia, iron–mitochondrial stress, hepcidin-mediated iron restriction, ferroptosis, neuroinflammation, glial reactivity, myelin stress, and BBB vulnerability provide biologically plausible but variably human-anchored bridges.

**Practice implications.** CBC, iron studies, renal function, and inflammatory context may help interpret AD blood biomarkers in selected older adults with multimorbidity, CKD, frailty, fatigue, nutritional vulnerability, or low or low-normal Hb. These data support conditional interpretive guidance and future trial design, not universal iron-panel screening or claims that correction prevents dementia.

**Abbreviations:** AD, Alzheimer disease; Aβ42/40, amyloid-β42/40 ratio; BBB, blood–brain barrier; BMI, body mass index; CBC, complete blood count; CKD, chronic kidney disease; CSF, cerebrospinal fluid; GFAP, glial fibrillary acidic protein; Hb, hemoglobin; NfL, neurofilament light chain; PET, positron emission tomography; p-tau217, phosphorylated tau 217.

## 5 Mechanistic bridges: how hematopoietic nutritional phenotypes may relate to brain vulnerability and biomarker readings

Consistent with Box 1, the mechanisms below are presented as biologically plausible, variably human-anchored bridges rather than proof that anemia or iron deficiency causes AD biomarker change or dementia progression.

### 5.1 Impaired oxygen delivery, cerebral vulnerability, and metabolic stress

The brain operates with high oxidative demand and limited metabolic reserve, so impaired oxygen delivery is best viewed as a vulnerability state, not a purely hematologic abnormality; in AD-related settings, hypoxia most plausibly amplifies metabolic stress, vascular dysfunction, and downstream proteinopathy-sensitive pathways without constituting a disease-specific mechanism on its own (123). Chronic cerebral hypoperfusion adds a sustained low-flow component, linking reduced perfusion to oxidative stress, endothelial injury, blood–brain barrier strain, and white-matter vulnerability in experimental cerebrovascular models (124). Human hemodynamic data make this entry point clinically relevant, as older adults with amnestic MCI show higher cerebrovascular impedance and lower normalized cerebral blood flow than cognitively normal peers (125). Associations between plasma biomarker profiles, cerebral perfusion, and brain structure in AD cohorts further suggest that perfusion state shapes the tissue milieu in which neurodegenerative signals are expressed, although these data do not establish a direct biomarker-specific mechanism (126).

Experimental mixed-dementia models extend this bridge by reporting that chronic cerebral hypoperfusion aggravates amyloid and tau pathology through impaired glymphatic transport involving aquaporin-4 and vascular endothelial growth factor pathways (127). Direct hypoxic stress also converges on tau-related biology, with hypobaric hypoxia inducing tau protein alterations in experimental systems (128). The HIF-1 response captures the ambivalence of this pathway: it can support short-term adaptation to low oxygen, yet persistent or dysregulated hypoxic signaling may also contribute to amyloid precursor protein processing, tau phosphorylation, and neuroinflammatory shifts (129). Together, these data position impaired oxygen delivery as an upstream vulnerability that may amplify cerebral metabolic stress, oxidative injury, and neuroinflammatory tone rather than as an AD-specific mechanism in itself (123, 130).

### 5.2 The iron–mitochondria–energy failure axis

Iron is integral to neuronal energy metabolism, as heme groups and iron–sulfur clusters support electron transfer, respiratory-chain enzymes, aconitase activity, and redox buffering; disturbed iron handling can therefore threaten mitochondrial respiration before overt anemia becomes evident (131). In AD, this vulnerability intersects with pre-existing defects in oxidative phosphorylation, ATP production, calcium handling, mitophagy, mitochondrial dynamics, and electron-transport-chain function (132, 133). Experimental work supports this link: TfR1 knockdown alleviated iron overload and mitochondrial dysfunction in AD-derived induced pluripotent stem cell neural differentiation models through interaction with GSK3β (134), and presenilin mutation studies in *Caenorhabditis elegans* suggest that AD-related biology can disrupt iron homeostasis and promote ferroptosis-mediated neurodegeneration (135). Here, ferroptosis is treated as a convergent redox–bioenergetic vulnerability that integrates iron dyshomeostasis, lipid peroxidation, antioxidant depletion, ROS burden, iron–sulfur cluster stress, and membrane injury, rather than as a separate explanatory pathway (136–138).

### 5.3 Hepcidin, chronic inflammation, and functional iron deficiency

Chronic inflammation recasts iron deficiency as a problem of availability, not total iron quantity. Hepcidin integrates inflammatory and renal signals, suppresses ferroportin-dependent iron export and intestinal uptake, and can produce iron-restricted erythropoiesis even when storage markers appear preserved or difficult to interpret (139). In older hospitalized patients, inflammatory status was associated with reduced intestinal iron absorption, offering a geriatric example in which functional restriction may emerge before the phenotype is fully captured by Hb, low ferritin, or a conventional anemia label, particularly when acute-phase biology obscures ferritin interpretation (140). Within dementia biomarker studies, plasma hepcidin appears most useful as a contextual marker, with its signal centering on iron–inflammation biology and VaD differentiation, not on a stand-alone role as a core AD blood biomarker (141). Human effect-modifier evidence supports that placement: in virally suppressed people with HIV, hepcidin altered the relation between anemia, erythrocyte indices, and neurocognitive performance, showing that it is more than a parallel laboratory value disconnected from clinically relevant brain outcomes (142).

**Figure 2:**
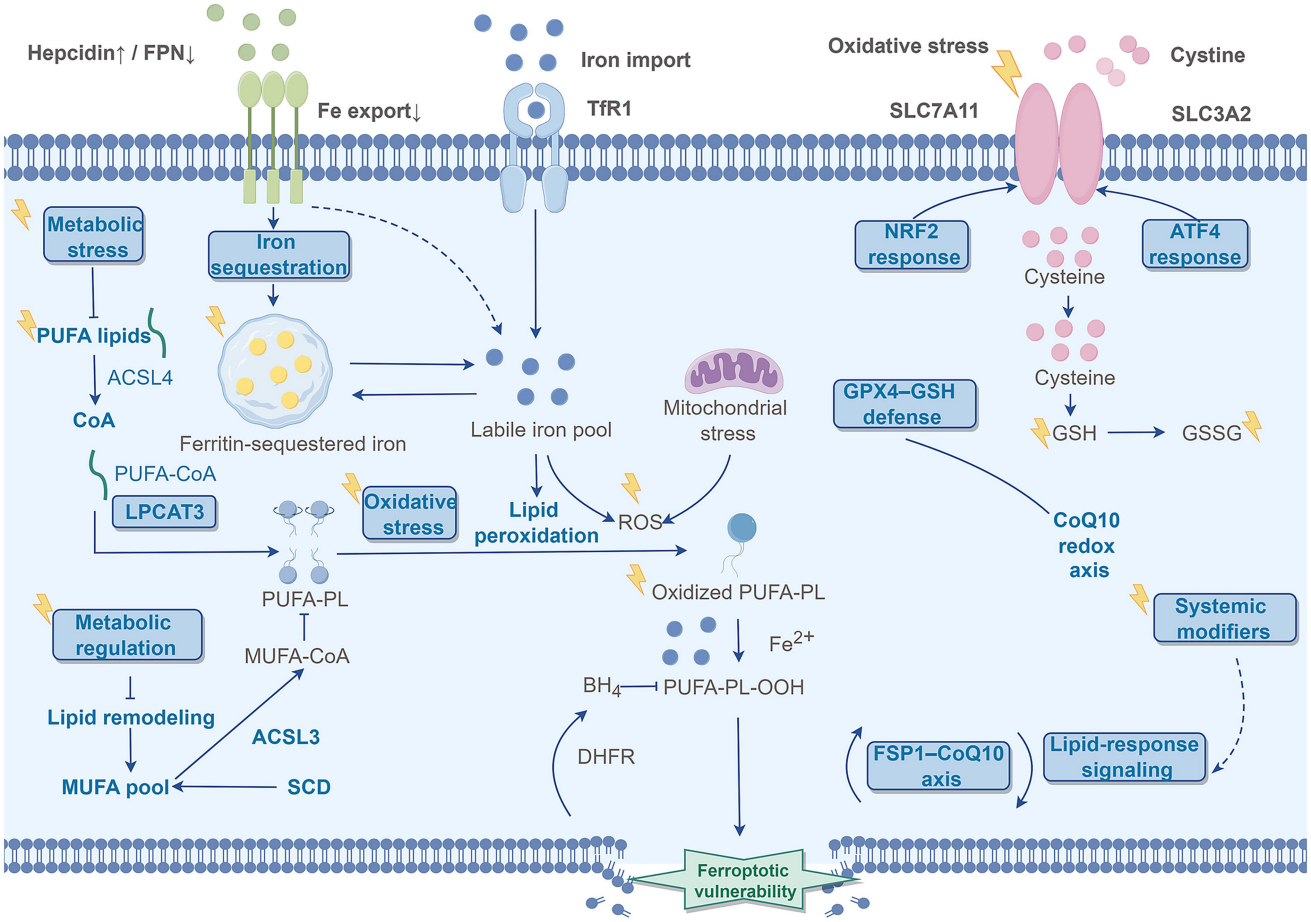
Hepcidin–iron–ferroptosis axis linking functional iron restriction to cellular vulnerability. Created by Figdraw.com.

Reports of local hepcidin upregulation in AD brain tissue, together with ferroportin downregulation and iron accumulation, suggest that the hepcidin–ferroportin axis may contribute to brain iron retention as well as peripheral erythropoietic restriction (143). Links between hepcidin, cytokine increases, and disrupted iron homeostasis in AD and Down syndrome dementia further place this axis within neuroinflammatory iron biology (144, 145). These data place hepcidin–ferroportin signaling as a systemic and potentially local entry point for iron restriction or retention; downstream glial, myelin, and blood–brain barrier (BBB) vulnerability is considered in the tissue-level discussion below (Figure 2).

Chronic inflammation and renal dysfunction may increase hepcidin signaling, reduce FPN-mediated Fe export, and promote iron sequestration, labile iron stress, mitochondrial ROS generation, PUFA-phospholipid peroxidation, and ferroptotic vulnerability. SLC7A11/SLC3A2-mediated cystine import, the cysteine–GSH–GPX4 pathway, NRF2/ATF4 stress responses, the FSP1–CoQ10 axis and CoQ10 redox signaling, BH4–DHFR defense, and MUFA remodeling are shown as simplified, parallel counter-regulatory defenses. Dashed arrows indicate indirect regulatory effects, and blunt-ended lines indicate inhibitory or protective effects. This schematic is intended as a mechanistic bridge rather than evidence that hepcidin, iron restriction, or ferroptosis alone causes AD. The figure was created using Figdraw. AD, Alzheimer disease; ACSL3, acyl-CoA synthetase long-chain family member 3; ACSL4, acyl-CoA synthetase long-chain family member 4; ATF4, activating transcription factor 4; BH4, tetrahydrobiopterin; CoA, coenzyme A; CoQ10, coenzyme Q10; DHFR, dihydrofolate reductase; Fe, iron; Fe2+, ferrous iron; FPN, ferroportin; FSP1, ferroptosis suppressor protein 1; GPX4, glutathione peroxidase 4; GSH, reduced glutathione; GSSG, oxidized glutathione; LPCAT3, lysophosphatidylcholine acyltransferase 3; MUFA, monounsaturated fatty acid; MUFA-CoA, monounsaturated fatty acyl-CoA; NRF2, nuclear factor erythroid 2-related factor 2; PUFA, polyunsaturated fatty acid; PUFA-CoA, polyunsaturated fatty acyl-CoA; PUFA-PL, polyunsaturated fatty acyl phospholipid; PUFA-PL-OOH, polyunsaturated fatty acyl phospholipid hydroperoxide; ROS, reactive oxygen species; SCD, stearoyl-CoA desaturase; SLC3A2, solute carrier family 3 member 2; SLC7A11, solute carrier family 7 member 11; TfR1, transferrin receptor 1.

### 5.4 Glial reactivity, axonal injury, myelin stress, and BBB homeostasis

These upstream pressures become biologically legible in brain tissue when translated into astrocyte–microglia reactivity, since glial states can remodel the local environment in which amyloid, tau, vascular injury, and neuroaxonal stress are expressed (146). In Alzheimer mouse models, astrocytes and microglia participate in C1q-dependent elimination of excitatory and inhibitory synapses, supporting a pathway that turns glial activation into circuit-level injury (147). Oligodendrocytes belong in the same picture, with AD-related oligodendrocyte states increasingly linked to myelin maintenance, stress adaptation, lipid handling, and white-matter vulnerability (148). Human and mouse subcellular proteomic and imaging studies further identify the myelin–axon interface as a vulnerable site in AD, making axonal injury and myelin stress inseparable components of the same tissue-level readout (149).

Myelin dysfunction may also amplify proteinopathy, as experimental AD models link disturbed myelin integrity to enhanced Aβ deposition, which argues against treating myelin loss purely as a late downstream consequence (150). BBB biology adds a vascular-inflammatory layer: human AD studies associate BBB dysfunction with AD pathology, cognitive impairment, and neuroinflammation, bringing the BBB and neurovascular unit into the same vulnerability field as glial and myelin injury (151). A cellular entry point for this link comes from pericyte-mediated endothelial dysfunction, with Aβ oligomer exposure reported to induce cerebral vasculopathy through pericyte–endothelial mechanisms (152). These glial, axonal, myelin, and barrier pathways provide a tissue-level context for plasma GFAP, NfL, and related neurodegenerative readouts, although current human evidence does not yet directly connect anemia or functional iron deficiency to these responses (153).

### 5.5 Boundary conditions of the mechanistic evidence

These mechanistic links should therefore be interpreted as graded evidence. Even improved mouse models do not fully reproduce the sequence from hematopoietic nutritional phenotypes to vascular injury and circulating biomarkers (154). Human data provide partial anchors: anemia has been linked to smaller brain volume (155), brain iron in AD-signature regions to atrophy and cognitive change (156), and CSF vascular injury markers to tau pathology and cognitive decline (157). By contrast, peripheral immune findings remain heterogeneous, and current immune, BBB, and choroid plexus literature more strongly supports broad AD vulnerability than an anemia- or iron-restriction-specific causal chain (158–160). Thus, the mechanistic evidence supports biological plausibility and study design rather than direct human causal inference.

## 6 From evidence to practice: risk stratification, biomarker interpretation, and future intervention studies

### 6.1 When CBC and iron studies may help contextualize biomarker testing in memory clinics and geriatric settings

In memory clinics and geriatric cognitive services, CBC can be considered as part of the pre-biomarker clinical context rather than as a stand-alone prerequisite for every AD blood biomarker test. This framing is consistent with implementation recommendations that place history taking, cognitive assessment, routine bloodwork, comorbidity review, and medication review within the same patient journey used to determine whether an AD blood test is interpretable, actionable, and appropriately routed to specialist confirmation (2). CBC alone may be insufficient when anemia, low-normal Hb, microcytosis, frailty, fatigue, nutritional risk, occult blood loss, or clues to malabsorption are present, because consensus recommendations support ferritin- and TSAT-based evaluation whenever iron deficiency is clinically plausible, even before a formal anemia threshold is crossed (44). Reduced eGFR or established CKD provides a particularly strong rationale for concurrent CBC and iron studies, given that CKD anemia guidance already treats CBC, reticulocyte count, ferritin, and TSAT as core baseline tests and renal dysfunction is itself a recognized modifier of biomarker interpretation (42). Implementation studies reinforce the value of establishing this context early in selected patients, with clinicians across specialties emphasizing that readiness for blood biomarkers depends not only on assay access, but also on interpretive support, performance data, and clarity about how results will influence referral, confirmatory testing, or treatment planning (161).

This conditional approach is especially relevant in old-age psychiatry, frailty-oriented services, and oldest-old primary-care settings, where limited follow-up laboratory access or incomplete PET/CSF confirmation can make systemic context important for interpreting AD biomarker results (162, 163). Simultaneous CBC and iron studies are most reasonable when multimorbidity, CKD or inflammatory burden, nutritional vulnerability, fatigue, frailty, unexplained anemia, or low or low-normal Hb could affect referral, PET/CSF testing, counseling, treatment eligibility, or follow-up planning. The aim is to improve interpretation and identify relevant systemic contributors, not to promote indiscriminate iron screening or a universal pre-biomarker rule (164).

### 6.2 How to interpret elevated p-tau217, GFAP, or NfL in the presence of anemia or iron deficiency

Interpretation of elevated p-tau217, GFAP, or NfL in anemia or iron deficiency should avoid two extremes: dismissing the result as a hematologic artifact and treating it as AD-equivalent without context (72). For p-tau217, evidence that kidney function, BMI, and anemia can affect cutoffs supports assay-specific and context-aware thresholding, with %p-tau217 potentially less sensitive to systemic dilution, clearance, or hematologic effects than raw concentration (18). The dominant analyte should guide next steps: p-tau217 carries the strongest AD-pathology relevance, GFAP is a glial and context-sensitive signal, and NfL reflects broader neuroaxonal injury and is less AD-specific (165, 166).

Accordingly, p-tau217-dominant results should still raise concern for AD biology, especially when outside the intermediate zone and consistent with the clinical syndrome. Disproportionate NfL elevation should prompt broader evaluation for vascular disease, non-AD neurodegeneration, frailty, systemic illness, or acute/subacute neurological injury, whereas GFAP-dominant elevation should be interpreted as a context-sensitive glial-response signal (167, 168). In practice, interpretation should first define hematologic and iron status, renal function, inflammatory context, BMI, assay platform, cutoff zone, and pretest probability; then classify the biomarker pattern; and finally select PET, CSF, specialist review, repeat testing, or longitudinal follow-up according to intended use and decision impact (169, 170). Box 2 provides a concise algorithm.

Box 2 **Conditional clinical algorithm for CBC, iron studies, and AD blood biomarker interpretation.**

**Step 1:** Use hematologic and iron data when they may affect decisions.

Review CBC, ferritin, TSAT, renal function, and inflammatory context when AD blood biomarker results will influence referral, PET/CSF confirmation, counseling, treatment eligibility, or follow-up, especially in older adults with multimorbidity, CKD or reduced eGFR, inflammatory burden, nutritional vulnerability, fatigue, frailty, unexplained anemia, microcytosis, or low or low-normal Hb.

**Step 2:** Define the context before interpreting the biomarker pattern.

Record anemia status, iron phenotype, eGFR, inflammatory context, BMI, assay platform, cutoff zone, and pretest probability; then classify the result as p-tau217-dominant, GFAP-dominant, NfL-dominant, or mixed.

**Step 3:** Interpret according to the dominant pattern.

A p-tau217-dominant pattern should continue to raise concern for AD biology, particularly when outside the intermediate zone and consistent with the clinical syndrome; anemia, CKD, altered BMI, or inflammation may modify cutoff confidence but should not by itself justify dismissing the result. A GFAP-dominant pattern is best read as a glial and context-sensitive signal, whereas an NfL-dominant pattern should prompt broader evaluation for neuroaxonal injury, vascular disease, non-AD neurodegeneration, frailty, systemic illness, or acute/subacute neurological injury. Mixed patterns should prompt systemic-context review and, when decision-relevant, specialist review, PET/CSF confirmation, repeat testing, or longitudinal follow-up.

**Step 4:** Manage gray-zone or discordant results as unresolved evidence.

Options include repeat testing with the same calibrated assay, PET/CSF confirmation, longitudinal biomarker follow-up, or reassessment after anemia, CKD, inflammation, or iron-restricted states are clarified. Correction of anemia or iron deficiency should not be taken as evidence that AD biomarker trajectories are normalized or that dementia is prevented.

**Abbreviations:** AD, Alzheimer disease; BMI, body mass index; CBC, complete blood count; CKD, chronic kidney disease; CSF, cerebrospinal fluid; eGFR, estimated glomerular filtration rate; GFAP, glial fibrillary acidic protein; Hb, hemoglobin; NfL, neurofilament light chain; PET, positron emission tomography; p-tau217, phosphorylated tau 217; TSAT, transferrin saturation.

### 6.3 Anemia and iron deficiency as risk phenotypes, analytic modifiers, or both

At this stage, anemia and iron deficiency are best treated as phenotypes that may carry both risk and biomarker-modifier information rather than being assigned a fixed causal label. Cohort evidence links iron deficiency to incident dementia risk (96), while biomarker-variability studies show that Hb, kidney function, BMI, APOE, and other systemic factors can shift AD-related blood biomarker distributions (91). Community screening data similarly indicate that peripheral risk-factor clusters involving Hb, red-cell distribution width, and inflammation are associated with GFAP, NfL, t-tau, and related readouts before specialist diagnostic pathways are considered (171). Hepcidin findings support the relevance of iron–inflammation biology in mixed vascular and neurodegenerative contexts, without making hepcidin a stand-alone AD biomarker (141). Multiethnic cohort data linking lower hematocrit to incident dementia further support a prognostic dimension of erythroid vulnerability (172). Thus, anemia or iron deficiency may function as a risk phenotype, a modifier of biomarker interpretation, or both in the same patient; the unresolved translational question is whether correction changes biomarker trajectories or risk classification reproducibly and meaningfully (173, 174).

### 6.4 Future trials: testing whether correction of iron-related phenotypes alters biomarker trajectories or risk classification

Future trials should be designed to test, rather than assume, whether correcting anemia or iron deficiency produces interpretable change in AD fluid biomarker endpoints, which are now common in disease-modifying AD trials but have not yet been validated for nutritional phenotype correction (175). Enrollment should be phenotype- and risk-zone-based, and real-world p-tau217/p-tau181 performance in a Southeast Asian tertiary memory clinic offers a useful template for intended-use strata, amyloid-probability zones, and risk-reclassification endpoints that ask whether correction moves patients across practical decision thresholds (176). Serial biomarker endpoints must be interpreted with reference change values, as biological-variation estimates in healthy individuals show analyte-specific within-person variability in p-tau, GFAP, NfL, and related plasma measures that can render small pre-post differences biologically uninterpretable (177). Intended-use populations also need their own repeat-testing rules, since short-term variability in mixed memory-clinic cohorts can differ from that seen in healthy adults and should guide sampling interval, minimum meaningful change, and the credibility of trajectory endpoints (178).

Nutritional trials also require strict pre-analytical control. A pilot food-intake intervention showed postprandial shifts in several AD blood biomarkers, so fasting status, meal timing, draw time, and acute intake need protocolization before biomarker changes are interpreted as effects of iron or anemia correction (179). If one AD-specific trajectory endpoint has to be prioritized, plasma p-tau217 is the strongest candidate, as endpoint-modeling work suggests that p-tau217 can support smaller and more efficient AD trial designs, especially in amyloid-enriched or intermediate-amyloid populations (180). The trial-ready question is not whether iron repletion or anemia treatment can move a biomarker once, but whether a predefined intervention produces biomarker change that exceeds the reference change value (RCV), changes risk classification, alters downstream decisions, and informs the design of larger prevention or treatment-selection trials (181).

### 6.5 Research priorities and practical implications

Until such trials are available, specialized-care guidance provides an appropriate boundary for current practice: blood biomarkers should be used within intended-use diagnostic workups for patients with objective cognitive impairment, after clinical evaluation, routine bloodwork, review of systemic context, counseling, and consideration of confirmatory pathways (3). Research priorities should focus on calibrated implementation, including whether plasma biomarkers can triage anti-amyloid immunotherapy candidates, reduce unnecessary PET/CSF testing, preserve safe eligibility decisions, and maintain performance across diverse populations and locally validated thresholds (182, 183). Probability-based approaches, including Bayesian p-tau217 models and combinations with locally normed cognitive testing, offer practical templates for gray-zone management in real-world and resource-constrained settings (79, 184). In practice, adoption should be staged and context-aware, with hematopoietic phenotype, pretest probability, test purpose, and downstream decision impact considered when clinically relevant rather than treated as universal screening requirements (185). Practical implications for clinical translation are summarized in Table 5, and a conditional clinical interpretation algorithm is provided in Box 2.

**Table 5:** Clinical translation of phenotype-aware biomarker use.

Source	Question	Message	Caveat	Refs.
Policy implementation	How should testing enter care?	Clinical workup first; triage before confirmation	Not hematopoietic-specific	(2)
Specialized-care guideline	What performance is needed?	Separate triage and confirmatory thresholds	Low certainty; assay variability	(3)
Workflow study	Can plasma testing reduce PET/CSF?	Two-cutoff p-tau217 may reduce PET/CSF	Not anemia/iron-focused	(182)
Oldest-old primary care study	Can biomarkers aid triage?	Cognition plus biomarkers may aid triage	No PET/CSF or p-tau217	(163)
Mixed memory clinic cohort	Does p-tau217 need local validation?	Validate thresholds locally	Proxy ethnicity	(183)
Cutoff and cost study	When is context needed?	CKD/BMI/anemia may justify adjusted cutoffs	Hb-defined anemia; no iron panel	(18)
Bayesian implementation study	How should probability be used?	Convert pretest to posttest probability	Small; assay-specific	(79)
Low-education implementation study	How should cognition be paired?	Pair p-tau217 with local cognitive norms	Screening cohort	(184)
Southeast Asian memory clinic study	Which zones guide amyloid risk?	Low, intermediate, and high zones guide risk	Small amyloid-confirmed subset	(176)
Biological-variation study	What serial change is meaningful?	Use RCV before judging change	Healthy adults only	(177)
Short-term variability study	What repeat change is meaningful?	Use repeat-testing rules from intended-use cohorts	Small mixed cohort	(178)
Biomarker comparison study	Does hepcidin help interpretation?	Limited AD value; possible VaD signal with NfL	Not CBC/iron-panel study	(141)

Studies address implementation or interpretation, not intervention efficacy. AD, Alzheimer disease; BMI, body mass index; CBC, complete blood count; CKD, chronic kidney disease; CSF, cerebrospinal fluid; Hb, hemoglobin; NfL, neurofilament light chain; PET, positron emission tomography; p-tau217, phosphorylated tau 217; RCV, reference change value; VaD, vascular dementia.

## 7 Discussion and conclusion

### 7.1 Take-home messages

AD blood biomarkers now have direct clinical consequences, but they remain most useful when applied within intended-use populations and interpreted alongside clinical, cognitive, imaging, fluid, digital, and systemic information (186, 187). Within this pathway, anemia, absolute iron deficiency, and functional iron deficiency should be treated as separable hematopoietic nutritional phenotypes because iron biology intersects with oxygen delivery, mitochondrial energetics, myelin integrity, and neurodegenerative vulnerability in ways that Hb alone cannot capture (188).

These phenotypes are not substitutes for AD pathology, but they can improve integrated interpretation of p-tau species, GFAP, NfL, and related biofluid signals (189). The next priorities are to define pre-analytical handling, repeatability, serial-measurement rules, and clinically meaningful change, and then to test whether phenotype-aware strategies improve biomarker-guided decisions, risk classification, and trial design rather than merely producing one-time laboratory shifts (190, 191).

### 7.2 Why this framework matters for neuro-nutrition and dementia-prevention research

This perspective matters for neuro-nutrition because it moves the field beyond broad diet–cognition correlations toward phenotype-defined biology: anemia, absolute iron deficiency, and functional iron restriction can be examined in relation to mechanisms of brain aging instead of being hidden inside Hb labels (192). In the biomarker era, lifestyle and nutritional factors also belong within the interpretive context of plasma AD markers, as nutrition is increasingly considered alongside other modifiable exposures that may shape biomarker distributions or their clinical meaning (193).

For dementia-prevention research, the value is practical. Hematopoietic nutritional phenotypes may inform counseling, risk stratification, and trial design as measurable states rather than vague comorbidities (194). The next step is not uniform dietary advice but phenotype-defined nutrition studies that integrate laboratory iron status with cognitive outcomes and biomarker or neuroimaging readouts so that prevention hypotheses become directly testable (195).

## Glossary

AD: Alzheimer disease
Aβ: amyloid-β
Aβ42/40: amyloid-β42/40 ratio
APOE: apolipoprotein E
APS2: amyloid probability score 2
ATN: amyloid/tau/neurodegeneration
BBB: blood–brain barrier
BMI: body mass index
CBC: complete blood count
CKD: chronic kidney disease
CRP: C-reactive protein
CSF: cerebrospinal fluid
DSST: Digit Symbol Substitution Test
eGFR: estimated glomerular filtration rate
FPN: ferroportin
GFAP: glial fibrillary acidic protein
Hb: hemoglobin
IL-6: interleukin-6
MCI: mild cognitive impairment
NfL: neurofilament light chain
NHANES: National Health and Nutrition Examination Survey
PET: positron emission tomography
p-tau: phosphorylated tau
p-tau181: phosphorylated tau 181
p-tau217: phosphorylated tau 217
%p-tau217: percentage-based plasma phosphorylated tau 217
p-tau231: phosphorylated tau 231
RCV: reference change value
TRIAD: Translational Biomarkers in Aging and Dementia
TSAT: transferrin saturation
t-tau: total tau
VaD: vascular dementia
WHO: World Health Organization
